# Detailing the role of parametric T_1_- and T_2_-mapping for differentiation of acute and chronic myocardial infarction

**DOI:** 10.1186/1532-429X-17-S1-P9

**Published:** 2015-02-03

**Authors:** Florian von Knobelsdorff-Brenkenhoff, Marcel Prothmann, Matthias A Dieringer, Ralf Wassmuth, André Rudolph, Wolfgang Utz, Julius Traber, Andreas Greiser, Thoralf Niendorf, Jeanette Schulz-Menger

**Affiliations:** 1 Siemens Healthcare, Erlangen, Germany; 2 Berlin Ultrahigh Field Facility, Max-Delbrueck-Center, Berlin, Germany; 3 Cardiology, ECRC, Charité University Medicine Berlin and HELIOS Clinics, Berlin, Germany

## Background

T_1_- and T_2_-mapping in myocardial infarction may be helpful to discriminate acute from chronic stages (AMI, CMI). This study examines the role of T_1_- and T_2_-mapping versus today's T_1_- and T_2_-weighted imaging standard along with interpretation modes for discrimination of AMI from CMI.

## Methods

Eight male patients with acute ST-elevation myocardial infarction underwent CMR at 3T (Siemens Verio) acutely and after >3 months. Imaging techniques included: T_2_-weighted imaging, late enhancement (LGE) as well as T_2_-mapping (3 single-shot SSFP-images), native T_1_-mapping (MOLLI, 11 single-shot SSFP-images), and T_1_-mapping 10min after 0.2mmol gadobutrol using non-product sequences. Image analysis included: 1) Visual assessment: Five independent readers assessed the presence (yes/no) of an infarct-like myocardial lesion. 2) Quantitative assessment per segment: Myocardial T_2_- and T_1_-relaxation times were determined for every segment and correlated to LGE. 3) Quantitative assessment per pixel: Based on reference T_2_- and T_1_- relaxation times, abnormal pixels were identified and correlated to LGE.

## Results

1) T_2_-weighted images showed an infarct-like lesion in 82.9% of AMI and 27.8% of CMI, LGE in 95.0% / 100%, T_2_-map in 69.2% / 35.0%, native T_1_-map in 86.8% / 57.5%, and post-contrast T_1_-map in 95.0% / 91.9%. 2) The pattern of segmental abnormalities of T_2_- and T_1_-relaxation times in infarcted segments compared to remote myocardium was not consistent for a confident diagnosis of AMI and CMI. 3) Pixelwise threshold-based analysis of T_2_- and T_1_-maps exposed infarcted regions in the myocardium in AMI and CMI. The presence of T_2_-abnormalities in the chronic state and the classification of remote pixels as abnormal limited its diagnostic value. The figure shows the various images and maps as well as segmental relaxation times of one representative patient.

**Figure 1:**
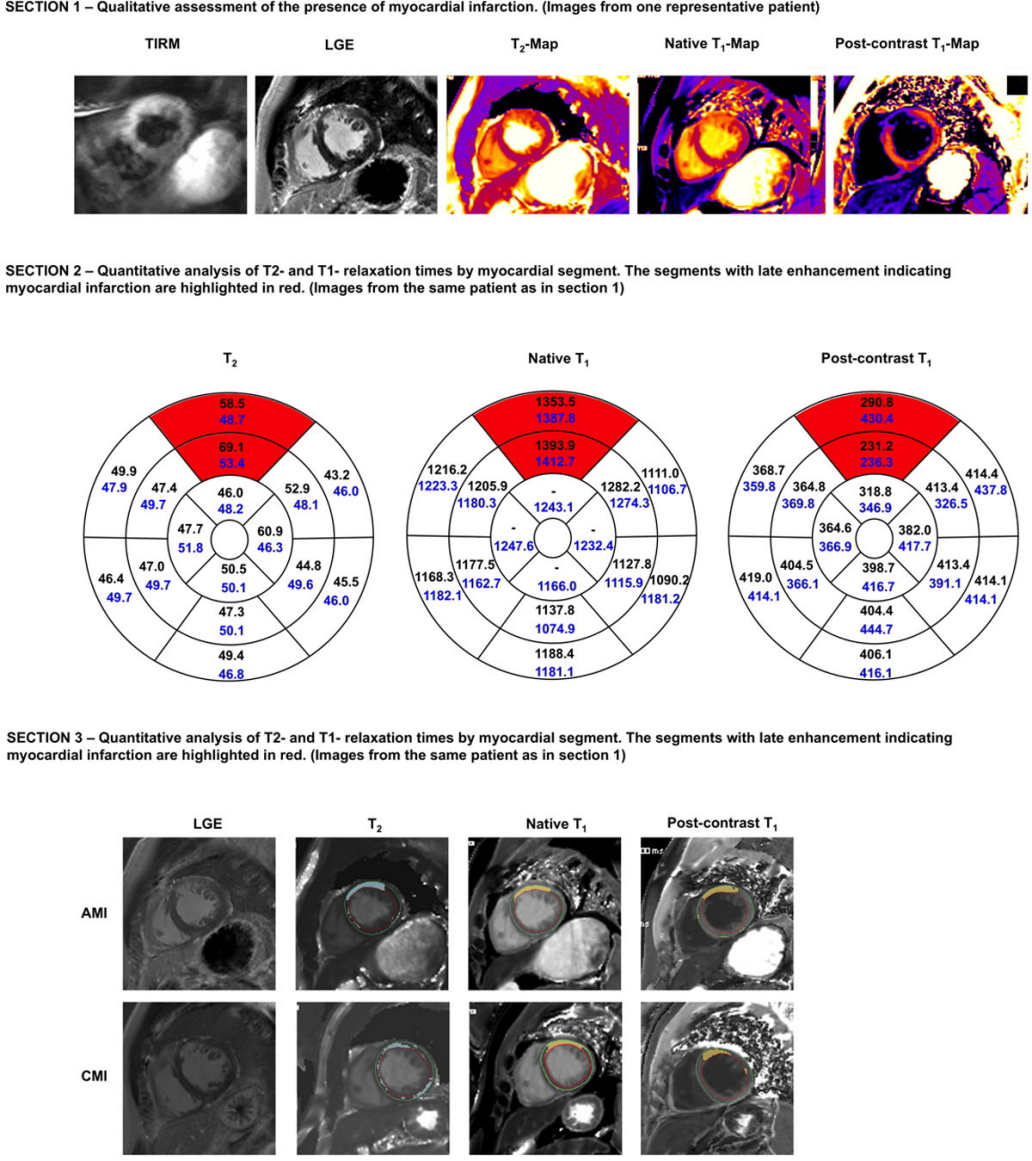


## Conclusions

Referring to the studied mapping sequences, pixelwise analysis of T_2_- and T_1_-maps based on predefined thresholds that separate normal from abnormal was the most promising approach to read maps, whereas visual assessment and segmental analysis of T_2_- and T_1_-maps were less favorable. The discrimination of AMI and CMI is not facilitated using the tested T_2_- and T_1_-maps.

## Funding

This project was supported by the Else Kröner-Fresenius Stiftung (Bad Homburg, Germany) (2010_A70).

